# Supplementary material for: Suicide and Associations with Air Pollution and Ambient Temperature: A Systematic Review and Meta-Analysis
Source: Int J Environ Res Public Health. 2021 Jul 20;18(14):7699. doi: 10.3390/ijerph18147699 (PMC8303705; doi:10.3390/ijerph18147699)
Supplement: Supplementary file 1 [file ijerph-18-07699-s001.zip › ijerph-1266366-supplementary.pdf]

Table S1. Search strategies.

Database	Search Terms
**Pubmed**	Term 1:<br>(“air pollution” or “air pollutant” or “air pollutants”) AND (“cohort” or “case control” or "epidemiolog*") AND (“suicide”)<br>Term 2:<br>(“air pollution” or “air pollutant” or “air pollutants” or “PM10” or “PM2.5” or “particulate”) AND (“suicide”) AND (“cohort” or “case control”)<br>Term 3:<br>(“air pollution” or “air pollutant” or “air pollutants”) AND (“observation*” or "epidemiolog*") AND (“suicide”)<br>Term 4:<br>(“particulate”) AND (“observation*” or "epidemiolog*") AND (“suicide”)<br>Term 5:<br>("air pollution" or "air pollutant" or "air pollutants") AND ("suicide")<br>Term 6:<br>(“air temperature” or “ambient temperature” or “temperature”) AND (“cohort” or “case control” or "epidemiolog*" or “observation*”) AND (“suicide*”)
**Scopus**	Term 1:<br>(“air pollution” or “air pollutant” or “air pollutants”) AND (“cohort” or “case control” or "epidemiolog*") AND (“suicide”)<br>Term 2:<br>(“air pollution” or “air pollutant” or “air pollutants” or “PM10” or “PM2.5” or “particulate”) AND (“suicide”) AND (“cohort” or “case control”)<br>Term 3:<br>(“air pollution” or “air pollutant” or “air pollutants”) AND (“observation*” or "epidemiolog*") AND (“suicide”)<br>Term 4:<br>("air temperature" or "ambient temperature" or "temperature" or "climate" or "weather") AND ("cohort" or "case control" or "epidemiolog*" or "observation*") AND TITLE-ABS-KEY("suicide*") AND (LIMIT-TO (DOCTYPE,"ar")) AND NOT INDEX (medline)
**Web of Science**	Term 1:<br>(“air pollution” or “air pollutant” or “air pollutants”) AND (“cohort” or “case control” or "epidemiolog*") AND (“suicide”)<br>Term 2:<br>(“air pollution” or “air pollutant” or “air pollutants” or “PM10” or “PM2.5” or “particulate”) AND (“suicide”) AND (“cohort” or “case control”)<br>Term 3:<br>(“air pollution” or “air pollutant” or “air pollutants”) AND (“observation*” or "epidemiolog*") AND (“suicide”)<br>Term 4:<br>(“particulate”) AND (“observation*” or "epidemiolog*") AND (“suicide”)<br>Term 5:<br>("air pollution" or "air pollutant" or "air pollutants") AND ("suicide")<br>Term 6:<br>ALL=(“air temperature” or “ambient temperature” or “temperature” or “weather”) AND ALL=(“cohort” or “case control” or "epidemiolog*" or “observation*”) AND ALL=(“suicid*”)

Table S2. MOOSE Checklist.

No.	Recommendation	Reported on Page No
	Reporting of background should include	
1	Problem definition	2-4
2	Hypothesis statement	4
3	Description of study outcome(s)	4-5
4	Type of exposure or intervention used	4, Table 1
5	Type of study designs used	4, Table 1
6	Study population	4, Table 1
7	Qualifications of searchers (eg, librarians and investigators)	4
8	Search strategy, including time period included in the synthesis and key words	4
9	Effort to include all available studies, including contact with authors	4
10	Databases and registries searched	4
11	Search software used, name and version, including special features used (eg, explosion)	NA
12	Use of hand searching (eg, reference lists of obtained articles)	4
13	List of citations located and those excluded, including justification	4-5
14	Method of addressing articles published in languages other than English	NA
15	Method of handling abstracts and unpublished studies	4
16	Description of any contact with authors	NA

17	Description of relevance or appropriateness of studies assembled for assessing the hypothesis to be tested	5
18	Rationale for the selection and coding of data (eg, sound clinical principles or convenience)	5
19	Documentation of how data were classified and coded (eg, multiple raters, blinding and interrater reliability)	5
20	Assessment of confounding (eg, comparability of cases and controls in studies where appropriate)	5-6
21	Assessment of study quality, including blinding of quality assessors, stratification or regression on possible predictors of study results	5-6
22	Assessment of heterogeneity	5
23	Description of statistical methods (eg, complete description of fixed or random effects models, justification of whether the chosen models account for predictors of study results, dose-response models, or cumulative meta-analysis) in sufficient detail to be replicated	5
24	Provision of appropriate tables and graphics	NA
25	Graphic summarizing individual study estimates and overall estimate	6, 14, 15
26	Table giving descriptive information for each study included	7
27	Results of sensitivity testing (eg, subgroup analysis)	14, 16, 17
28	Indication of statistical uncertainty of findings	17, 18, 19

NA=not applicable.

Table S3. Qualitative risk summary for the included studies.

Exposure	Positive Associations but not Significant	Significantly Positive Associations	Negative Associations but not Significant
Temperature	Dixon, 2018<br>Fernandez-Nino, 2018<br>Hiltunen, 2012<br>Luan, 2019<br>Maes, 1994<br>Salib, 1997<br>Yazra, 2020	Bando, 2017<br>Basagana, 2011<br>Barker, 1994<br>Basu, 2018<br>Deisenhammer, 2003<br>Grjibovski, 2013<br>Hiltunen, 2014<br>Hu, 2020<br>Merrill, 2019<br>Kayipmaz, 2020<br>Kim, 2011<br>Kim, 2016<br>Kim, 2019<br>Kubo, 2021<br>Lee, 2020<br>Likhvar, 2011<br>Muller, 2011<br>Page, 2007<br>Santurtún, 2020<br>Schneider, 2020<br>Sim, 2020<br>Williams, 2016<br>Zerbini, 2018	Fountoulakis, 2016<br>Makris, 2021
$PM_{2.5}$	Kim, 2018<br>Nguyen, 2021	Bakian, 2015<br>Liu, 2019	Astudillo-Garcia, 2019<br>Fernandez-Nino, 2018
PM_{10}	Astudillo-Garcia, 2019<br>Casas, 2017<br>Kim, 2010<br>Kim, 2018<br>Li et al. 2018	Kim, 2015<br>Lee, 2018<br>Lin, 2016<br>Szyszkowicz, 2010	Fernandez-Nino, 2018<br>Ng, 2016
O_3	Casas, 2017<br>Lee, 2018<br>Yang, 2019b	Kim, 2015<br>Nguyen 2021<br>Yang, 2019a	Astudillo-Garcia, 2019<br>Fernandez-Nino, 2018
SO_2	Kim, 2015<br>Kim, 2018<br>Szyszkowicz, 2010	Lee, 2018<br>Lin, 2016	Astudillo-Garcia, 2019<br>Fernandez-Nino, 2018<br>Kim, 2015<br>NG, 2016
NO_2	Kim, 2015<br>Kim, 2018	Bakian, 2015<br>Lee, 2018<br>Lin, 2016<br>Szyszkowicz, 2010	Astudillo-Garcia, 2019<br>Fernandez-Nino, 2018<br>NG, 2016<br>Thilakaratne, 2020
CO	Fernandez-Nino, 2018<br>Kim, 2015	Lee, 2018<br>Szyszkowicz, 2010	Thilakaratne, 2020

Text S1. Sensitivity analysis for meta-regression analysis.

In sensitivity analysis, we applied meta-regression analysis for the Gross Domestic Product (GDP) per capita (currency US$) and the Purchasing Power Parity (PPP) per capita (US$) obtained from the World Bank [1]. As the World Bank's classification of countries by income level was based on GNI per capita and no particular cut-off references were found for income classifications of GDP per capita and PPP per capita, we used the mean values of these indexes among the study countries to classify higher- and lower-income groups in the meta-regression analysis. For example, the cut-off at $30025 was used to classify higher- and lower-income groups based on GDP per capita. The RRs of suicide associations with an interquartile range (IQR) increase in the exposure factors are shown in Table S5. Results based on GDP per capita were robust to the RRs based on classifications by GNI per capita. RR of suicide associated with temperature was lower in higher-income countries (RR = 0.92, 95% CI: 0.87, 0.98) than the RR in lower-income countries (RR = 1.20, 95% CI: 1.14, 1.26), which was significantly different (p-value = 0.005). Likewise, RR was lower in higher-income countries than lower-income countries based on PPP per capita. For air pollutants, RRs tended to be higher in higher-income countries but the risk differences were not significant. In summary, income level was a significant effect modifier for the suicide-temperature associations, whereas it was not a significant effect modifier for the suicide risks associated with short-term exposure to air pollutants.

References

1. The World Bank GNI per capita, Atlas method (current US$) Available online: https://data.worldbank.org/indicator/NY.GNP.PCAP.CD (accessed on Apr 1, 2021).
